# Diagnostics barriers and innovations in rural areas: insights from junior medical doctors on the frontlines of rural care in Peru

**DOI:** 10.1186/s12913-015-1114-7

**Published:** 2015-10-05

**Authors:** Cynthia Fiorella Anticona Huaynate, Monica Jehnny Pajuelo Travezaño, Malena Correa, Holger Mayta Malpartida, Richard Oberhelman, Laura L Murphy, Valerie A Paz-Soldan

**Affiliations:** Universidad Peruana Cayetano Heredia, Universidad Peruana Cayetano Heredia, Av. Honorio Delgado, 430. SMP. Lima, Peru; Department of Global Community Health & Behavioral Sciences, Tulane University School of Public Health and Tropical Medicine, 1440 Canal Street, Suite 2301, New Orleans, LA 70112 USA; Department of Global Health Systems & Development, Tulane University School of Public Health and Tropical Medicine, 1440 Canal Street, Suite 1900, New Orleans, LA 70112 USA

**Keywords:** Health innovation, Diagnostics, Barriers, Rural health, Primary health care, Peru

## Abstract

**Background:**

Worldwide, rural communities face barriers when accessing health services. In response, numerous initiatives have focused on fostering technological innovations, new management approaches and health policies. Research suggests that the most successful innovations are those involving stakeholders at all levels. However, there is little evidence exploring the opinions of local health providers that could contribute with further innovation development and research. The aims of this study were to explore the perspectives of medical doctors (MDs) working in rural areas of Peru, regarding the barriers impacting the diagnostic process, and ideas for diagnostic innovations that could assist them.

**Methods:**

Data gathered through three focus group discussions (FGG) and 18 individual semi-structured interviews (SSI) with MDs who had completed their medical service in rural areas of Peru in the last two years were analyzed using thematic analysis.

**Results:**

Three types of barriers emerged. The first barrier was the limited access to point of care (POC) diagnostic tools. Tests were needed for: i) the differential diagnosis of malaria vs. pneumonia, ii) dengue vs. leptospirosis, iii) tuberculosis, iv) vaginal infections and cervical cancer, v) neurocysticercosis, and vi) heavy metal toxicity. Ultrasound was needed for the diagnosis of obstetric and intra-abdominal conditions. There were also health system-related barriers such as limited funding for diagnostic services, shortage of specialists, limited laboratory services and access to telecommunications, and lack of institutional support. Finally, the third type of barriers included patient related-barriers to follow through with diagnostic referrals. Ideas for innovations proposed included POC equipment and tests, and telemedicine.

**Conclusions:**

MDs at primary health facilities in rural Peru face diagnostic challenges that are difficult to overcome due to a limited access to diagnostic tools. Referrals to specialized facilities are constrained by deficiencies in the organization of health services and by barriers that impede the patients’ travel to distant health facilities.

Technological innovations suggested by the participants such as POC diagnostic tools and mobile-health (m-health) applications could help address part of the problem. However, other types of innovation to address social, adaptation and policy issues should not be dismissed.

## Background

In rural communities worldwide health care providers often face a number of challenges and barriers when trying to provide services. Major challenges in the provision of health services in rural settings include limited transportation and communication systems and infrastructure, shortages of health professionals, and restricted access to resources for diagnostics, prevention and curative purposes [1–3]. In the recent past, numerous initiatives have tried to address some of these major challenges by developing and implementing technological innovations such as lab-on-a-chip tests, telehealth and m-health [4, 5]. Other innovations to obtain more effective and equitable health systems have also been advocated for, including new management approaches and changes to health policies [6, 7].

The development of health innovations should be informed by the unique needs of the end users and/or potential beneficiaries [8]. However, there are gaps between the priorities and perspectives of the people who develop innovations and the end-users [9]. Stakeholders at different levels (including innovators, donors and governments) could benefit from a deep understanding of the health related challenges and needs in the target settings. Health providers can be important sources of information to guide this process [10–12]. Previous studies have assessed the perceptions of rural health professionals regarding the specific needs to improve health services at their own settings, including point of care equipment for diagnosis and therapeutics [13, 14], information and communication technology [15–18] and training needs to overcome clinical problems and socio-cultural issues [19, 20].

The aims of this study were to explore and describe the perspectives of medical doctors (MDs) working in rural areas of Peru regarding: 1) barriers impacting their clinical practice, specifically focused on the diagnostic process, and 2) ideas for diagnostic innovations (including tools or services) that could assist them. Limited access to diagnostic tools can have a direct impact on misdiagnosis and inadequate access to treatment; it can also frustrate health providers and reduce patients’ trust in the health system [21].

Health care services in rural areas of Peru are mainly provided by recently graduated MDs who are enrolled in the program called “SERUMS” (the Spanish abbreviation that stands for medical service in rural and marginalized urban areas) at primary health facilities. Thus, we focused on this specific group of MDs to address our research objectives.

The present study was conducted as part of the Framework Program for Global Health Innovation called Inter-American Training for Innovation in Emerging Infectious Diseases (IATIEID), held by Tulane University (USA). Through an interdisciplinary approach, the program’s aim is to foster the development of innovations to address critical health problems in resource-poor settings [11].

### The context

#### Rural populations in low- and middle-income countries

In low- and middle-income countries, the population is still predominantly rural (55 % of total population live in rural areas), with the greatest proportion of rural people living in South Asia and Sub Saharan Africa (69 % and 63 % respectively) [22]. In Latin America and the Caribbean, most countries are highly urbanized; however, many countries still have significant rural populations, including Peru [23]. Twenty two percent of the total population in Peru lives in rural areas [24].

#### Peru, health status and health care services

Peru is considered a middle-income country with a growing economy. However, close to a third of the total population (29 million) lives in poverty; poverty rates are highest among people living in remote rural areas [25]. Peru has three distinct geographic regions (the coast, the Andes highlands, and the Amazon basin), and each of these regions has unique challenges that impact their health and development indicators [26].

The majority of Peru’s population (60 %) lives in the coast (comprising 16 % of the Peruvian territory) and this population is predominantly urban. Urbanization has led the demographic and epidemiologic transitions to progress faster on the coast compared to other regions. There is a higher prevalence of chronic diseases in the coast compared to other regions. However, the prevalence of certain infectious diseases, such as HIV/AIDS and tuberculosis remains high in this region [27–29].

Rural, isolated communities are common in the Andes highlands. These communities are difficult to access due to the rugged mountains and limited road system. The population is predominantly poor (50 % poverty rate) and many work in agriculture and mining [23]. In the central Andes, the mining activity has become a public health problem, associated with subsequent heavy metals exposure and chronic respiratory diseases [25]. In the southern Andes, characterized by the intensely cold winter months, infectious respiratory diseases are the leading cause of death in children under five years [26].

Only 12 % of Peru’s total population lives in the tropical Amazon basin (which comprises 50 % of the Peruvian territory), and beyond a few rainforest cities, communities are predominantly rural [23]. Most of the villages are located around several river basins. Infectious tropical diseases (i.e. malaria, dengue, leishmaniasis) are frequent and during the winter months, respiratory diseases also become a major health problem [27].

In rural areas, health services are mainly provided by the Ministry of Health, through the Integral Health Insurance, the “SIS”. The objective of the SIS is to provide free health care to vulnerable populations living in poverty. However, the SIS has only 40 % coverage at national level; and coverage in rural areas is even more limited [28]. Moreover, the SIS benefits package only covers 25 % of the disease burden, so individuals still have high out-of-pocket expenses on complex care and medicines [29].

The rural health infrastructure is based on two types of primary health facilities: health posts and health centers. Health posts (HPs type I-1 and I-2) are located in the communities and the health centers (HCs) are usually located in a provincial or district capital [30]. HCs are better equipped than HPs in terms of health personnel and resources.

- HPs type I-1 offer only health promotion and prevention services, provided by a technician.
- HPs type I-2 have a physician who is responsible for ambulatory care for a number of diseases or conditions which do not require diagnostic tests.
- HCs are assigned at least two MDs and have a basic laboratory for a limited number of tests (cell blood count, hematocrit, basic immunology, pregnancy diagnosis, VDRL test (blood test for syphilis), basic microbiology, sputum smear test and basic biochemistry tests] [31].

Table 1 shows the general characteristics of the primary health facilities by category. Cases requiring more specialized care are referred to usually distant hospitals (not shown in the table).

**Table 1:** Characteristics of the participants’ primary health facilities, by category

Category	I – 1	I – 2	I – 3
Health facility in the MOH system	Health post	Health post with physician	Health center
Community health promotion	Yes.	Yes.	Yes.
Ambulatory consultation	No.	Yes.	Yes.
Physician	No.	General (usually serumista).	General or family doctor.
Other health professionals	Nurse technician and/or nurse and/or midwife.	Nurse, midwife and nurse technicians.	Nurse, midwife, dentist, laboratory and pharmacy technicians.
Laboratory facilities	No.	No.	Yes.
Diagnostic tools available.	None.	Pregnancy tests, hemoglobin strips, rapid tests for HIV and syphilis (only for pregnant women).	Equipment for sample collection and processing. Equipment and reagents for complete blood count and hematocrit, blood group, basic immunology, pregnancy diagnosis, rapid VDRL (blood test for syphilis), basic microbiology, baciloscopy, and basic biochemistry.
Delivery room	No.	No.	No.

As is the case globally [3], staffing of rural health facilities in Peru is a serious problem. Resources are heavily concentrated in the main cities in the coast, and communities in the highlands and in the Amazon basin are underserved [25]. One attempt to address the understaffed health facilities has been the implementation of the “SERUMS” plan to distribute and retain health workers in rural areas. SERUMS is a one-year remunerated program that takes place in the most rural, poor, and isolated areas of the country [32]. The rural population of the coast is small (out of the total rural population, 25 % live in the coast, 40 % in the Highlands, and 34 % in the Amazon basin) [33], thus the Highlands and Amazon basin host most (90 %) of the SERUMS vacancies for MDs. The SERUMS program is mandatory for all Peruvian health professionals who want to be affiliated with the public health employment system [32].

All health posts (HPs type I-2) and the majority of HCs in rural Peru are headed by MDs in the SERUMS program. Every year, around 2000 SERUMS doctors (known as “serumistas” in Peru) are appointed to these health facilities.

## Methods

Our approach follows an exploratory qualitative enquiry based on a convenience sample of MDs drawn from the SERUMS program. Data was gathered through focus group discussions (FGD) and semi-structured interviews (SSI).

### Participants

All participants were MDs who had completed the SERUMS program in rural areas, in either the Amazon basin or the Highlands region, in the last two years, and who were currently residing in the capital city of Lima. Participants were recruited at three educational centers where former serumistas receive training for their specialization examination, which is the next step in their medical career. After receiving a verbal description of the study at the end of one of their courses, the MDs were invited to participate. We sought participants who had performed their SERUMS in the Highlands and Amazon regions (where 90 % of the SERUMS posts are placed due to needs of these very rural and remote regions) as well as in the two types of health facilities to ensure a diversity of experiences (maximum variation sample). However, we did not exclude any MD interested in participating.

There were a total of 30 participants: 12 participated in three focus groups (with four participants in each) and 18 participated in the SSI. There were 17 men and 13 women aged 26–30 years. Sixteen participants had their SERUMS posts in the Highlands and 14 in the Amazon basin. None of the participants had worked in the Coast. Twenty had performed their SERUMS in a HP and 10 in a HC.

### Data collection techniques

The FGD and SSI were conducted in October and November 2013, in a private location close to the educational centers where the participants were recruited. The FGD lasted about 90 min and the SSI lasted around 60 min. Topics covered in both the FGD and the SSI were: i) barriers encountered in the diagnostic process of diseases or conditions that were particularly difficult to manage in their settings, and ii) suggestions for innovations that could improve the diagnosis process and clinical management generally.

FGD were facilitated by a team of four researchers: two researchers co-facilitated, and two observed and took detailed notes. The same four researchers conducted the SSI individually.

In addition to digital audio-taping of the FGD and SSI, detailed notes were taken and all written materials (i.e., flip charts used during the FGD) were retained as part of the notes. As notes were finalized for each of the FGD and SSI, the digital audio-recording was played to ensure all important messages were captured. The FGD and SSI were conducted in Spanish, the mother tongue of all study participants and researchers. Data was gathered until no new information was emerging.

### Methodological observations

We conducted both FGD and SSI concurrently, expecting these two methods would complement each other. We expected that the FGD would allow us to explore a wide range of general perceptions regarding diagnostics barriers and ideas for innovative solutions, and that the different participants might be stimulated by others’ responses. The SSI would allow us to explore certain issues with more depth, by having more time to discuss individual’s personal experiences.

### Data analysis

Both the FGD and SSI quotes were transcribed in Spanish and then translated into English by the same researchers who conducted the FGD and SSI (who are all bilingual). The interviewees were identified by a pseudonym between parentheses. A thematic analysis approach was used, in which we analyzed and reported data based on main themes within our two main topics of interest: barriers and ideas for innovations. The analysis was stratified by region (i.e., Highlands vs. Amazon) and the type of health facility where the serumistas were posted.

In the coding process, all the transcripts were read to develop a list of codes based on topics that emerged, as well as our topics of interest. Then, the transcripts were coded based on themes. In this process, several codes were further developed based on new findings, and transcripts were re-read to ensure that any new codes had been applied throughout.

### Ethics

This study was approved by the Biomedical IRB at Tulane University, New Orleans, USA and the Review Board at Universidad Peruana Cayetano Heredia, Lima, Peru. Verbal consent was obtained from all the participants.

## Results

Four key themes were identified from the FGD and SSI. These were:

i.) Lack of tools to address challenging types of diagnostic problems, ii) health system-related barriers to the diagnostic process, iii) patients’ barriers in following through with diagnostic referrals, and iv) ideas for technological innovation to enhance the diagnostic process.

### Lack of tools to address challenging types of diagnostic problems

Serumistas described various types of difficulties they faced when trying to diagnose specific diseases without the access to diagnostic tools at their health facilities. These diseases varied based on the region where they were posted. Although serumistas posted to HCs had more diagnostic resources than those posted to HPs, both groups expressed similar diagnostic challenges.

Serumistas posted to facilities in the Amazon region discussed difficulties making differential diagnoses between respiratory infections, malaria and other diseases that presented with acute febrile syndrome such as dengue and leptospirosis. These diseases share striking clinical similarities that complicate the diagnoses. Two serumistas working at different HCs in the Amazon commented: *“Once I had a pediatric patient presenting with signs of pneumonia and I prescribed antibiotics. But since the patient had not recovered after some days, I suspected it could be malaria. If I had had x-rays or any similar tool I could have diagnosed correctly that it was not pneumonia and I would have started him on anti-malaria treatment”* (Gio).

*"In January 2013, we had many patients who came with jaundice, fever, myalgia, and we assumed it was a dengue outbreak, although we did not have any diagnostic test on hand to ensure that. After a month we received the results of the lab tests that were completed in Lima, and found out that only 10 % of cases were dengue, the others were leptospirosis. By that time, we had already managed the cases as dengue and we were lucky that all cases evolved well but the clinical management would have been substantially improved if we had had a point of care (POC) diagnostic device”* (Garo).

Amazonian serumistas also reported frequent difficulties in diagnosing vaginal infections and cervical pre-cancerous or cancerous processes. Participants reported that this was due to a lack of resources for taking and analyzing samples to perform the Pap smear test. The equipment and resources needed for this test were only available at hospitals.

Serumistas posted to the Highlands expressed concern in assessing people’s potential exposure to heavy metals related to activities such as mining (i.e., lead, arsenic) and the use of agricultural pesticides (i.e., selenium) in certain provinces. This was another condition that serumistas found impossible to assess on site. Only a few laboratories in the capital city of Lima conduct analyses for heavy metals (i.e., Graphite furnace atomic absorption) in biological samples.

One serumista posted to a HP in a Highlands province noted the population experienced a high prevalence of taeniasis and he expressed a great need for POC diagnostic tests to evaluate suspected cases of neuro-cysticercosis.

Serumistas posted to departments in both the Highlands and the Amazon reported problems with tuberculosis (TB) diagnosis. Serumistas at HPs had no means by which to diagnose suspected TB cases. Serumistas at HCs had the resources to collect sputum samples for baciloscopy analysis, but faced another type of problem. They said it was very difficult to collect a complete and proper sputum sample because it required that patients visit the HCs at least three times, but most patients dropped out after the first visit.

All serumistas reported that obstetric problems were very difficult to assess and manage without an ultrasound machine. Gio did not have access to one for the entirety of his rural service. *"Over the period of one year, a total of 900 pregnant women attended my health facility and I needed an ultrasound [which I did not have] for prenatal control, diagnosis of obstetric complications, uterine rupture, miscarriages, and fetal deaths"* (Gio).

Ultrasound was also required to make the differential diagnosis between acute non-surgical vs. surgical abdominal conditions, such as the difference between appendicitis, which requires an expedited surgical procedure, vs. cholecystitis, which requires a deferred surgery.

### Health system-related barriers to the diagnostic process

**S**erumistas reported that barriers related to the lack of diagnostic resources were magnified by other health system-related barriers such as: i) the lack of funds for further diagnostic tests, ii) the shortage of specialists at referral facilities, iii) deficiencies of the laboratory services (unreliable and poorly equipped), iv) the limited access to telecommunications and v) lack of institutional support and opportunities for training.

As the public health insurance (“SIS” program) does not cover diagnostic services or expenses related to non-emergency transfers, most serumistas described patient referrals as ethically complicated decisions. They had to choose between referring a patient onward, requiring effort and cost for a potentially unnecessary procedure or keeping her/him at home or at the primary health facility, with potentially dangerous consequences. One serumista expressed: *“Once I had a patient with acute abdominal pain. I suspected it could be appendicitis but as the case was not clear, it was difficult for me to decide if I should refer him to the hospital or wait for a while. What if I sent him to the hospital and made him spend lots of money when the case was not that serious? I knew about many cases where patients ended up protesting that doctors made them waste money”* (Gabo).

The shortage of specialists (who work on average 15 days a month) at referral facilities means long waiting lists for appointments, exacerbating this dilemma. One serumista expressed: ‘*“At the beginning I used to send pregnant women to the hospital to have an ultrasound. Even if they reached the hospital at 5:00 am and were the first in line, the only thing they got was an appointment to return a month later. No one wanted to return and in the process, they lost confidence in me for making them waste their time”* (Juo).

Some serumistas who had worked at HCs expressed concerns about the skills of the laboratory personnel and the quality of the laboratory supplies. At least five serumistas reported experiences where erroneous laboratory results led to wrong clinical management: *“I had some problems when a laboratory technician came to work at my health center. As she had no previous experience, she gave us many false positive results for malaria. We only realized this when at the end of the month the confirmatory tests came from the city hospital. We had already given unnecessary treatment to some patients”* (Elo).

The low coverage of telecommunication services and the lack of guidance and training opportunities were also sources of great frustration for most serumistas. In many locations there was no telephone signal, making it difficult to arrange for referrals or consults with more experienced doctors outside the serumista’s site. And when the cell phone signal was available, most serumistas had to pay for the calls themselves. One serumista explained: *“In order to get a hospital referral for a patient I had to call from my own cellphone and due to the poor signal the process took ages. Sometimes I felt I only needed to make a consultation with a senior colleague but this was also complicated. I had to call the hospital and describe the case to a nurse or receptionist and asked for the colleague to call me back. If the nurse was a good person, she gave me the doctor’s private number and I called him again from my own cell phone”* (Migo).

The limited access to internet was also a barrier to access any literature or to participate in online courses for further professional development.

### Patients’ barriers in following through with diagnostic referrals

Serumistas stated that they often had to make referrals because of the lack of diagnostic tools at their health facilities. However, most referrals were not completed as patients were reluctant to be transferred for additional diagnostic tests or for a specialist consultation: “*The issue is that patients do not want to be transferred at all, so we are forced to handle the cases anyway, otherwise patients could die”* (Migo).

Most serumistas explained that these communities had other priorities that took precedence over health matters. One serumista remarked: *“People were reluctant to leave their communities even when they were strongly advised to travel and get hospital assistance for their own sake. They usually argued that although they felt sick, they could not risk leaving their herding on their own or lose one day of work”* (Ale).

Another serumista described a potentially tragic scenario that was difficult for her to manage: “*Once I suspected that a two-year old patient had pneumonia. I told his mother to take him to the hospital so he could have an X-ray to confirm if it was pneumonia and receive proper care. The mother did not accept arguing that she did not have money. Even when I offered to pay for the trip, she said: If he has to die, let him die here; just do whatever you can here”* (Rota).

The serumistas thought that access issues (i.e. transportation, costs) and the low quality services at referral facilities also affected patients’ decisions about referrals.

### Ideas for technological innovations to enhance the diagnostic process

Serumistas mostly talked about POC diagnostic tests, telehealth and m-health technologies.

#### POC diagnostic tests

One of the most frequently suggested innovations was ultrasound equipment adapted to rural settings to diagnose obstetric, abdominal and pulmonary diseases or conditions (instead of X rays). Serumista Cea commented: “*We would need a device with special software to help imaging interpretation for rural doctors who have not received any special training like us. If the software was good enough, it could also be used by nurses or the technician in charge of the most isolated HPs*” (Cea).

Juo added: “*If we could incorporate the ultrasound to a tablet or another similar sized-electronic device, it would be easy to carry to isolated villages or to make home consultations when the patients are not able to come to the health facility”* (Juo).

Other ideas included: i) multiplex tests designed for the differential diagnosis of dengue, pneumonia, malaria, leptospirosis and other diseases with similar clinical features, ii) a technique to collect a single sample of sputum for TB diagnoses given that *“patients come just once to leave their samples and rarely return to leave the following two”* (Jea), iii) a portable device to measure heavy metals in a range of biological samples, iv) a rapid test for the diagnosis of neuro-cysticercosis, and v) an automated microscope-based system that could differentiate among different parasites, to prevent potential erroneous laboratory results related to the lack of highly trained/ experienced laboratory personnel.

#### Telehealth and m-health technologies

Most serumistas reported that they had actually practiced some kind of m-health through peer networks: *“I used to send pictures of my patients that I took with my cellphone to my colleagues that were closer to the city so they could ask a specialist for advice. This is something that almost every serumista did in exchange for favors”* (Gio).

They thought that the implementation of a formalized mobile-based system to perform consultations with specialists could be very helpful to improve the referral system: *“It would be great to have a system that would allow serumistas to present difficult cases to specialists working in city hospitals in order to get advice”* (Joo). Another serumista proposed potential uses of m-health beyond that of clinical diagnosis: *“M-health could be useful to obtain information in real time, for instance, to know if there are available beds in the hospital”* (Ivo).

However most serumistas aware of the local conditions, doubted that such technology could be implemented effectively. One serumista who had had the opportunity to work in a province selected for a telemedicine pilot project run by the government in the Highlands, commented: “*There are a lot of things apart from the electronic system itself that such programs must achieve in order to succeed, such as training the whole health staff, promoting its use among communities, establishing a platform of infrastructure and personnel for maintenance and monitoring, among others. I really do not think our current health system will be able to implement all these things successfully”* (Alo).

Finally, serumistas discussed the potential impact of their suggested POC tests. The ultrasound imaging output produced in real time could help patients to *“see their disease”* (Elo), thus enhancing their understanding of the clinical manifestations of their diseases and reinforcing or increasing their trust in the public health service. Similarly, using telehealth or m-health applications to substitute consultations with specialists could help mitigate the shortage of personnel, accessibility and other barriers related to hospital referrals. However, serumistas pointed out that such systems would also have its limitations: “*Certainly it is not the same as evaluating the patient in person. Much information would be missed if only seeing the patient through a screen and receiving only somebody else’s perception of the patient status”* (Elo).

## Discussion

Three main findings emerged which merit discussion at the local and global context, given the similar challenges rural communities face worldwide: i) rural doctors face diagnostic challenges that are difficult to overcome due to the limited access to resources and other health system-related barriers, ii) patients’ barriers to following through with referrals exacerbates diagnostic challenges, and iii) integrated innovations, including new technologies, and social and policy strategies could enhance the diagnostic process.

### Limited access to diagnostic tools and other health system-related barriers

The serumistas face numerous diagnostic challenges that are often associated with the geographical region, for instance, differentiation between malaria and pneumonia in the Amazon region. To overcome the diagnostic challenges, serumistas need specific tools that are often not available to them.

This lack of resources in remote, rural health facilities is a problem globally. According to Strasser [3], a common factor shared by rural health systems in many countries is the deficit in their Primary Health Care (PHC) services.

In most low- and middle-income countries, primary health facilities often lack enough resources or pathways to meet the community’s immediate health problems [3].

In rural Peru, PHC is often provided in health facilities (HPs and HCs) with limited infrastructure and equipment that are mainly intended to offer promotional and preventative services. The current organization of the health services and allocation of resources in Peru [34] mandates that doctors heading rural primary health facilities should only manage a limited number of diseases or conditions that do not require auxiliary diagnostic tests. All other cases should be referred to more specialized health facilities [35], typically in distant locations. However, this formal description of roles and functions is not necessarily reflected in the actual activities. As we found, serumistas are often faced with patients who expect them to be able to treat more serious diseases or conditions onsite, which requires specific diagnostic tools.

A similar scenario is faced by practitioners in rural health posts worldwide with evidence showing that primary care practitioners are often called to cross the primary-secondary interface of health care in order to address the patients’ health care needs [13]. Studies in South Africa and Canada have reported that the more rural the area, the more likely it is that a general practitioner will find themselves engaged in complex care that requires more than the basic tools with which they are equipped [36, 37].

Serumistas also identified other barriers to the diagnostic process such as costs, laboratory and personnel capacity, and communications. Similar issues affecting the delivery of care in rural areas have been largely documented in both low- and high-income countries [36–39]. However, the initiatives to address these problems are scarce.

### Patients’ barriers to following through with referrals exacerbates diagnostic challenges

There were various explanations provided by the serumistas to explain patients’ lack of follow through with referrals they receive to higher level care, such as long waiting times (including needing to go in person to get an appointment and often having to return on a different day), and perceived poor quality of care. The serumistas also reported that they thought that the communities do not trust the public health services because of previous negative experiences, and some expressed that, from their point of view, the community members might prioritize activities of daily living (or survival) over following up with health issues. Research in other countries, including high-income countries such as New Zealand and Canada, reveal a similar pattern of underutilization of health care in rural populations [3]. One study looking at sociological aspects of rural communities has pointed out that rural communities, especially those with an indigenous background, have a strong connection with their family and their land, which is their source of sustenance [40]. Taking care of both their family and land is their first responsibility and being away from home implies great suffering. Therefore, the sicker they (or their dependents) feel, the less likely they are to agree to be transferred to a distant health facility [12]. Beyond cost issues and family obligations, this could explain the reluctance of individuals to long stays in hospitals or other health facilities that some of the serumistas observed in their communities.

### Integrated innovations could enhance the diagnostic process

Most serumistas perceived that the standard diagnostic tools would still not be adequate for the rural facilities due to the high costs, limited infrastructure, and lack of specialized personnel. This perception is consistent with repeated calls for more appropriate diagnostic technology that is viable for resource-poor settings globally [25].

The PHC approach proposed in the 1978 Alma-Ata Declaration advocated for the implementation of socially acceptable technologies to make health care universally accessible [25]. Unfortunately, there are still several existing “gold standard” diagnostic tests that are extremely complex to perform in resource-poor settings and their cost is often prohibitive for poor populations [41].

In response to these challenges, many research and donor agencies have been proposing and facilitating funds for new diagnostic technologies for global health. Some of these initiatives include the Grand Challenges Canada, the Bill & Melinda Gates Foundation, and the Innovative financing to shape markets for HIV/AIDS, malaria and tuberculosis (UNITAID).

However, it is not always clear whether the technologies produced are actually the ones that health care providers would use or need most often. [9, 42]. In order to prevent such gaps, several stakeholders, including research and donor organizations and public health officers in each country, should gain deeper insights into the major diagnostic challenges presented in different settings, new diagnostic technologies required, and primary facilitators and barriers for the use and scale-up of new technologies [9]. Furthermore, Pai et al. [43] have proposed that all these issues should be integrated into a global framework for POC testing development and implementation.

We enquired about innovations that could be useful to address some of the major diagnostic challenges the serumistas faced. Serumistas proposed a number of POC tests and ideas for the use of telehealth and m-health technologies that in fact, constitute important trends in global health technology development.

From the serumistas’ perspectives, the impact of developing these ideas into workable innovations that are low-cost, user-friendly and equipment-free would not only aid diagnosis and treatment, but also could help to address issues of cost, capacity, accessibility and shortage of personnel. By reducing the burden on patients associated to referrals and enhancing the patients’ understanding of the disease, there is also a subsequent increasing trust in the health system. A systematic review of primary care clinicians’ attitudes towards POC blood testing in Europe and Australia reported similar benefits. The use of POC testing helped patients to be more convinced, reassured and more satisfied with the MD decisions, compared to if no test had been used, leading to enhanced communication and an improved relationship between clinicians and patients [44–46].

Likewise the use of m-health technology offered economic and social benefits to rural communities in Kenya by reducing health expenditures, and even in some cases creating jobs for community health workers [47].

Concerns about potential drawbacks of these technologies also exist from the doctors’ side; there is a risk of over-reliance on diagnostic POC tests, undermining of clinical expertise, and over-testing [48, 49].

#### Technological innovations are not enough

Systemic interventions that take into account social, adaptation and policy innovations have been widely advocated [6, 50]. These types of innovations can directly help health providers/the health system to cope with social and cultural barriers and enhance the widespread adoption of new technologies. Some of the documented adaptations by rural health providers are focused on integrating community resources; for instance, providers consulting community leaders or elders regarding cultural issues, participating in community events, and integrated additional community resources [51, 52]. Similarly, one participant of this study described his own uncommon approach to treat patients in his SERUMS community by integrating traditional and Western medical practices. He felt this approach helped him to enhance his relationship with the community and increase the overall compliance with medical procedures. Related strategies have been reported in previous studies. In Sub-Saharan Africa, the use of humanitarian approaches that include community appreciated- non-harmful practices have helped to increase cultural acceptability of maternal health services [50, 51]. In Alaska and New Mexico, health care providers in small communities have incorporated the use of natural healers in their communities and other adaptation strategies to the cultural styles of their patients [48].

Modifying approaches to health care and engaging with communities, however, is not likely to occur at large scale without enabling policy, and the support of the health and education system in general [52].

### Strengths and limitations

This study is the first that we know of that investigates the perceptions of rural health care providers in Peru, regarding the main barriers related to the diagnostic process, at primary health facilities. This information could help guide the development and implementation of health innovations that can enhance the provision of services in rural resource-poor settings. This was an exploratory study with a modest sample of MDs. The fact that the participants were mostly young, had little working experience, and had not lived in rural areas prior to the SERUMS posting could have likely limited their capacity to perceive a more comprehensive picture of the barriers related to the diagnostic process. Their limited professional experience could have also limited their capacity to suggest innovations that could address the identified barriers. Nevertheless, we believe that the MDs fresh perspective, and their extended and challenging experiences during the SERUMS program allowed them to provide powerful insights that have been conveyed in this study.

The self-selection bias from the MDs enrolled in the study could be another limitation, implying that our findings might only have reflected the perceptions of the particularly frustrated group of MDs. However, it is generally known and there are public statistics showing that health personnel in rural areas of Peru work in very poor conditions which likely lead to frustration and dissatisfaction [53]. The potential bias of interpreting the participants’ quotes differently from how they were intended was partly addressed by conducting a verification of the themes and interpretations by all the authors.

## Conclusions

MDs at primary health facilities in rural Peru face diagnostic challenges that are difficult to overcome with limited access to diagnostic tools onsite and other barriers within the health system. Referrals to specialized facilities are constrained by deficiencies in the organization of health services and by barriers that impede the patients’ travel to distant health facilities

Suggestions for technological innovations such as POC diagnostic tools, telehealth and m-health applications in rural Peru raised by our participants could help address part of the problem. Indeed, the priorities and concerns of rural front-line clinicians should be taken into consideration by global and local initiatives to forge innovation.

However technological innovations are not the only answer. Technological innovations should be envisioned as scientifically sound, affordable, and easy to use tools that are part of a wider program that includes social and policy innovation, to achieve more effective and equitable health systems. Further research of our group will investigate the main themes encountered in this study by using quantitative methods in a larger sample of rural health care providers.

## Abbreviations

WHO: World Health Organization
M-health: Mobile health
MD: Medical doctor
USA: United States of America
IATIEID: Inter-American Training for Innovation in Emerging Infectious Diseases
HIV: Human immunodeficiency virus
AIDS: Acquired immunodeficiency syndrome
HP: Health post
HC: Health center
FGD: Focus group discussion
SSI: Semi structured interview
POC: Point of care
PHC: Primary health care
UNITAID: Innovative financing to shape markets for HIV/AIDS, malaria and tuberculosis
VDRL: Venereal disease research laboratory
